# Fluid Bolus Therapy in Pediatric Sepsis: Current Knowledge and Future Direction

**DOI:** 10.3389/fped.2018.00308

**Published:** 2018-10-25

**Authors:** Ben Gelbart

**Affiliations:** ^1^Paediatric Intensive Care Unit Royal Children's Hospital, Melbourne, VIC, Australia; ^2^Murdoch Childrens Research Institute, Melbourne, VIC, Australia; ^3^The University of Melbourne, Melbourne, VIC, Australia

**Keywords:** fluid therapy, pediatric, septic shock, sepsis, volume responsiveness

## Abstract

Sepsis is a leading cause of morbidity and mortality in children with a worldwide prevalence in pediatric intensive care units of approximately 8%. Fluid bolus therapy (FBT) is a first line therapy for resuscitation of septic shock and has been a recommendation of international guidelines for nearly two decades. The evidence base supporting these guidelines are based on limited data including animal studies and case control studies. In recent times, evidence suggesting harm from fluid in terms of morbidity and mortality have generated interest in evaluating FBT. In view of this, studies of fluid restrictive strategies in adults and children have emerged. The complexity of studying FBT relates to several points. Firstly, the physiological and haemodynamic response to FBT including magnitude and duration is not well described in children. Secondly, assessment of the circulation is based on non-specific clinical signs and limited haemodynamic monitoring with limited physiological targets. Thirdly, FBT exists in a complex myriad of pathophysiological responses to sepsis and other confounding therapies. Despite this, a greater understanding of the role of FBT in terms of the physiological response and possible harm is warranted. This review outlines current knowledge and future direction for FBT in sepsis.

## Introduction

The worldwide burden of sepsis in pediatric intensive care in terms of morbidity and mortality remains high and is a key healthcare priority (1–3). Fluid bolus therapy (FBT) has long been the central component of resuscitation of children with sepsis (4). The role of FBT is to improve the circulating volume, cardiac output and mitigate circulatory dysfunction and organ hypoperfusion. It is the recommended forefront therapy of international pediatric and adult consensus guidelines in high-income and low-income settings (5–8). The emergence of evidence demonstrating harm associated with FBT has led to a re-evaluation of its role in sepsis resuscitation.

Data supporting current pediatric sepsis guidelines are limited. Recommendations in relation to FBT have been based on small case control studies and animal data, mostly from two-three decades ago (9–11). Few randomized controlled studies exist. The most recent 2017 ACCM/PALS guidelines recommend that 20–60 ml/kg should be administered, titrated to clinical signs of shock and discontinued at shock resolution or fluid overload (5). Fluid resuscitation (FR) for refractory shock and assessment of response is recommended within 15 min to which adherence has proven difficult (12, 13). The past two decades has seen large multicentre studies targeting optimal fluid composition (14, 15), goal directed therapy (16–18), fluid restrictive protocols (19–23) as well as a pivotal study of FBT vs. no FBT in African children with sepsis (24); all contributing to the current landscape.

Clinicians aim to identify patterns of circulatory dysfunction in septic shock that include myocardial dysfunction, systemic vasodilatation, and hypovolaemia (25). This generally relies on clinical examination and non-invasive haemodynamic parameters. The challenge of investigating whether interventions that independently, or in combination with others, improve outcomes or cause harm may prove difficult (26). The complexity of assessing one component of a suite of interventions to address a multifaceted pathophysiological process will require carefully designed studies. Yet, in the face of many unanswered questions and associated harm, the imperative to investigate the role of FBT in sepsis exists.

This review outlines the current understanding of the role of FBT in children with sepsis and recent research direction.

## Epidemiology of sepsis, patterns of fluid resuscitation and outcomes in children

The global burden of sepsis and septic shock is high in pediatric intensive care units (PICUs) with prevalence studies suggesting mortality rates ranging from 6 to 25% (1, 27). Temporal trends suggest that although prevalence may be increasing, severe sepsis mortality might be declining (2). In PICUs in ANZ, the prevalence of sepsis and septic shock is 2.9 and 2.1% respectively and accounts for over a quarter of PICU deaths (28). In the US, an observational study of septic shock in children indicated that a third of deaths occur early (1–3 days) with a high proportion occurring in previously healthy children (29). The most common causes of death being refractory shock followed by secondary organ dysfunction. In ANZ, Schlapbach et al. demonstrated that 50% of deaths from sepsis occurred within 48 h and that predictors of death unsurprisingly relate to presence of markers of multi-organ dysfunction (30). Pediatric sepsis mortality in low income countries range widely (31) due to definitions of shock, disease specific and population specific factors as well as differences in intensive care resources.

There are very few large scale epidemiological or randomized studies of FBT in pediatric septic shock (32). Several pediatric observational studies of the resuscitation phase of sepsis have mostly been small single center studies and compare survivors and non survivors (33, 34), those with or without shock (12), or protocol adherence (13, 35, 36). Those reporting outcomes with volume or timing of FBT show varying results. Paul et al. for instance, showed that those who received 60 ml/kg of FBT within 60 min in the emergency department had a 57% shorter hospital length of stay than children who did not (13). Whether this relates to early recognition and implementation of a range of interventions such as early appropriate antibiotics or FBT is unclear. An audit of pediatric sepsis management from the United Kingdom showed that the initial median volume of FBT prior to intensive care is 50–60 ml/kg (12) suggesting alignment to current guidelines in the initial phase of sepsis management.

A large US adult study of the interaction between fluid administration on day 1 and mortality from sepsis showed increased severity adjusted mortality and cost for each liter above 5L; in the presence of shock, mechanical ventilation or both (37). Leisman et al. however, showed in an observational cohort study of adults with sepsis that less time to initiation of FBT reduced hospital mortality, ICU admission, mechanical ventilation duration and length of ICU and hospital stay. These were adjusted for measures of organ dysfunction, patient source and antibiotic administration (38). The inherent limitations in this study preclude a causal relationship however of note, no difference in hospital mortality was observed for volume of FBT until >35 ml/kg was administered where mortality was increased. Whether improved survival relates to timely recognition of sepsis, improved bundle delivery remains unclear. There are a paucity of similar pediatric data associating outcomes with fluid resuscitation.

## Pathophysiological aspects of sepsis and response to FBT

The pathophysiology and haemodynamic patterns in septic shock are complex, dynamic and not easily determined clinically. The pathophysiological hallmarks of septic shock are cytokine and nitric oxide mediated inflammation, activation of the coagulation cascade, manifesting as myocardial, endothelial, and organ dysfunction (25). Therapeutic targets in the acute management of septic shock are fundamentally aimed at matching oxygen delivery to demand by improving cardiac output for which the key targets are macrovascular.

### Circulatory markers of shock

Clinical signs of septic shock such as tachycardia, hypotension, impaired skin perfusion, while readily identifiable and indicative of shock, are difficult to rely upon to indicate hypovolaemia or volume responsiveness. Yet these are commonly the triggers or targets available to clinicians in the first hours of pediatric sepsis management. More advanced tools such as echocardiography or invasive haemodynamic monitoring can assist in deciphering myocardial dysfunction from a hyperdynamic circulation as well as volume responsiveness, however even these, as static measures lack predictive accuracy (39). Their availability are not always available outside of the intensive care environment or during anesthesia. Both dynamic and static measures of volume responsiveness are unreliable in children (40, 41) and volume responsiveness as a concept, is somewhat arbitrary. Respiratory variation in aortic blood flow velocities appear better predictors of volume responsiveness in children (42, 43) more so than systolic pressure and pulse pressure variation (44) but this is in the context of ventilated children.

Blood pressure and heart rate are the most highly rated clinical signs among pediatric intensivists (45), ED physicians (46), adult intensive care physicians (47, 48). Yet blood pressure measured non-invasively are prone to underestimation. A study of over 50,000 concurrent non-invasive and invasive BP measurements showed that NIBP has a poor positive predictive value of 58% for hypotension meaning over treatment of low blood pressure is possible (49). The quality and volume of the peripheral and central pulses are also critical signs that rely on experience to elucidate and interpret accurately.

Capillary refill time (CRT) is a simple bedside test universally regarded as a marker of inadequate perfusion and dehydration in children and a specific adjunct sign of shock (50, 51). In children with septic shock in intensive care it relates weakly to stroke volume index (52) but commonly relied upon to determine the responsiveness to FBT. A study of adults with septic shock suggest good interrater reliability, good correlation with lactate and SOFA score and 14 day mortality (53) and trials of tissue perfusion-guided therapy (including CRT) on outcomes in sepsis, have ensued (54).

Several studies describe the haemodynamic patterns in children with septic shock, often following an initial dose of FBT commonly referred to as “fluid refractory shock.” Deep et al. showed distinct patterns of “cold' (predominantly reduced myocardial function, vasoconstricted) and “warm” (predominantly hyperdynamic, vasodilatory) shock amongst 36 children with community acquired and hospital acquired sepsis with early and sustained abnormalities in haemodynamic values (55). An Indian study in two PICUs showed that in 48 children who had received 40 ml/kg of FBT the continued presence of both “warm” and “cold” shock based on clinical and echocardiographic indices, was also accompanied by the transformation or evolution over time indicating the dynamic nature of the circulatory disturbance (56). Others have also defined the clinical and haemodynamic phenotypes using a pulmonary artery catheter (57). Ceneviva et al. showed persistence of shock following FBT (60 ml/kg) in over a third of patients. The distribution of shock patterns were low cardiac index (CI) (58%), high CI/low systemic vascular resistive index (SVRI) (20%) and low CI/low SVRI (20%) (57). Similarly, in another small cohort of children with “fluid refractory shock,” non-invasive cardiac output monitoring demonstrated marked differences in physiological patterns between those with catheter related sepsis and community acquired pneumonia (58).

Clearly a spectrum of circulatory phenotypes exists, overlap and evolve in the initial stages of septic shock in children. The ability of clinicians to recognize these entities early and repeatedly on the basis of predominantly clinical signs, commence therapies and use clinical, biochemical, echocardiographic, and perhaps microcirculatory markers to judge response to therapy outlines the complex nature of sepsis resuscitation and teasing out the role of one therapeutic intervention.

### Sepsis and the microcirculation

Imaging of the microcirculation to measure the number of perfused capillaries and capillary density can assess the microvascular response to sepsis and therapy. It is performed in intensive care patients with a sublingual camera using side-stream dark field video-microscopy of the sublingual circulation. Alterations in the microvasculature include reduction in capillary density and microvascular blood flow (59, 60). Microvascular dysregulation that occurs in sepsis include altered rheology of red blood cells, impaired regional vascular autoregulation, activation of coagulation and arteriovenous shunting (60). The microvasculature plays an independent role in tissue perfusion and oxygenation that may not be influenced by macrovascular alterations (61). In an observational study of 18 pediatric sepsis patients, persistent reduction in microcirculatory flow in the first few days of sepsis was associated with mortality (59). In relation to response to FBT, few animal studies using intra-vital microscopy and video imaging of the microcirculation have shown both improvement as well as persistence in microcirculatory dysfunction with FBT (62) whereas a small observational study in adults with sepsis showed that fluid responders (determined by a 5% increment in stroke volume), increased capillary density and flow to FBT compared to non-responders (63). Near-infrared spectroscopy is another non-invasive modality that can assess tissue oxygenation at the bedside and may have a place in assessing the microcirculatory manifestations of septic shock and response to therapy. The clinical utility of measures of the microcirculation in the resuscitation phase of septic shock remains to be seen.

Perhaps, in time, assessment of the phenotypic subtypes of septic shock may extend beyond clinical signs and haemodynamic measured and include genetic markers (64). Until then, the fundamental principles of using a constellation of clinical signs and haemodynamic monitoring in the resuscitation of septic shock with an emphasis on repeated assessments of response to therapy, will remain.

## Physiological responses to FBT

Pharmacodynamics assessment of FBT in post-operative adults show that the maximal effect on cardiac output occurs at 1.2 mins in responders and the effect dissipates at 10 min (65). A systematic review of studies looking at haemodynamic responses also support the findings that increases in cardiac output following FBT is unsustained at 30 min (66). In healthy adult volunteers, rapid IV bolus of 30 ml/kg of 0.9% saline and 4% albumin lead to differences in effects on pulmonary mechanics, inflammation and cardiac preload (67). Specifically, those who received 0.9% saline had increased pulmonary oedema with an inflammatory component whereas those who received 4% albumin did not. In adults in an emergency department setting the 5% changes in HR and BP from baseline measured at 10 min post FBT were not sustained at 1 or 2 h (68). There are limited data on the pharmacodynamic effect of FBT in children. A recent small cohort study however, compared echocardiographic changes in the first 24 h following FBT and rehydration vs. rehydration alone in malnourished African children with gastroenteritis (69). There were heterogeneous effects on echocardiographic markers of stroke volume in the bolus group; more so when compared to the continuous rehydration group. Long et al. in a prospective observational study showed a transient increase in cardiac index (a product of heart rate and echocardiographic derived stroke volume per meter squared of body surface area; L/min/m^2^) 5 min following a fluid bolus that had dissipated by 60 min to a lower baseline than pre bolus (70). Observational studies such as these are limited by confounding factors but do reflect the reality in clinical practice. It also suggests that that minutely time intervals may be required to understand physiological effects of FBT in a more granular way. The duration, magnitude and dissipation of effect of FBT in children require further examination.

## Evidence base and guidelines for FBT in pediatric sepsis

The two recent editions of the Surviving Sepsis Campaign Guidelines (6, 71) and the ACCM PALS guidelines have not altered their recommendations relating to FBT in sepsis (5, 72). They recommend that 20 ml/kg boluses up to 60 ml/kg be administered in the first 15 min of resuscitation unless signs of fluid overload occur. The World Health Organization report on the management of critically ill children, in 2016 recommended that for the treatment of non-specific shock, 10–20 ml of crystalloid be administered between 30 and 60 min with an emphasis on repeated re-assessments (7). The foundation of these recommendations is largely based on limited human and animal data as well as expert opinion. One of the pivotal observational cohort studies from 1991 investigated the association of fluid administration and mortality in children with septic shock. Thirty four subjects were categorized by administered volumes of FBT in the first hour of septic shock; < 20 ml/kg, 20–40 ml/kg and more than 40 ml/kg (9). The study showed that those who received >40 ml/kg of FBT had improved survival compared to those who received < 20 ml/kg.

There are few randomized studies of FBT in children with septic shock in the context of intensive care resources. Three studies have compared a range of interventions such as fluid types, early inotrope and goal directed therapy with measured outcomes such as shock reversal, mortality, and intensive care resources (18, 73, 74). These studies included a total of 309 children and when systematically reviewed, there were no discernible difference in patient-centered outcomes (75).

The majority of studies of FBT in children relate to disease specific conditions such as malaria (76, 77), dengue fever (78–80), and meningococcal sepsis (81) limiting their broad applicability. Systematic review and meta-analysis of these studies (excluding the FEAST study) do not provide compelling evidence for a mortality benefit from FBT vs. no FBT or for different types of FBT (82).

## Fluid expansion as supportive therapy (FEAST) trial

The FEAST study, a RCT of FBT in over 3,000 Sub-Saharan African children with sepsis and impaired perfusion has been a pivotal study in generating interest in the potential harm from FBT. It showed that boluses of 0.9% saline or 5% albumin compared to maintenance fluid significant increased mortality at 48 h (RR 1.45; 95% CI 1.13-1.86; *p* = 0.003). The results were consistent across all pre-specified subgroups including malaria, anemia (hemoglobin concentration < 50 mg/l), coma and lactic acidosis (lactate >5 mmol/l). These results generated much interest and debate surrounding the role of FBT in high-income countries (83–85). The investigators assigned causes of death based on clinical features at presentation and concluded that cardiovascular collapse, as the terminal event, was the largest contributor to excess mortality as opposed to pulmonary or neurological failure (86). Important perspectives regarding this trial have been outlined (84, 87) but increasing interest in examining FBT has followed in both adults and children. The main limitations of these findings have been well articulated by Duke (87). Firstly, despite being a clearly unwell population of children, shock, defined by the WHO (7) as presence of cold peripheries and weak pulse, tachycardia and delayed capillary refill >3 s was not present in around 70% of participants. Secondly, the lack of availability of intensive care interventions limits the ability to respond to complications of fluid therapy and thirdly, the population studied may well have been at risk of adverse consequences of fluid therapy such as the presence of cerebral oedema, hyponatremia or excessive antidiuretic hormone secretion.

## Fluids and harm: fluid overload

Fluid accumulation in critical care is recognized as being associated with respiratory and renal morbidity as well as increased ICU Length of stay (LOS) and mortality. The degree to which FBT contributes to fluid accumulation in children is not well established. The association of fluid overload and harm is consistent in a broad spectrum of critically ill children including those following congenital heart disease surgery (88–90), acute kidney injury (91, 92), acute lung injury (93), children on ECMO (94), in a general PICU (95, 96), children with shock (97), and sepsis (98). However, fluid overload is defined, either by percentage of weight accumulation or percentage increase in daily cumulative fluid balance, the association stands in a dose dependant fashion (99). The downstream effects of FBT on fluid accumulation in children with sepsis is likely to represent one of many aetiological factors including non-resuscitation fluid, impaired clearance mechanisms, physiological responses such as SIADH and endothelial dysfunction. Furthermore, how fluid overload (commonly identified from the medical record by net change in fluid input and output) relates to organ oedema, organ perfusion and function, is not clear. Fluid administration is a key modifiable component of fluid accumulation and the impact on organ oedema and function requires further examination.

## Fluid restrictive resuscitation strategies

In response to the concern regarding harm from FBT, studies of restrictive fluid resuscitation have emerged to assess feasibility and safety of early inotrope based resuscitation strategies in adults, in high and low income countries (19, 20, 100). Two pediatric studies exist; one in the UK (23) and one in Canada (22). The UK study randomized 75 children with infections and clinical signs of shock after 20 ml/kg of FBT to either 10 ml/kg or 20 ml/kg per bolus for subsequent boluses. At the end of the 4-h study period the mean difference in FBT volume was −11.2 ml/kg (95% CI −16.6 to −5.8 mL/kg; *p* < 0.001). Roughly two-thirds received only 1 further bolus. There were no differences in hospital or PICU based outcomes. The authors concluded that lower than expected severity of illness precludes conduct of a larger study. The Canadian study aims to determine whether early vasoactive therapy, compared to usual fluid resuscitation practice (up to 60 ml/kg of isotonic fluid) reduces time to shock reversal and organ dysfunction. Adult data have shown that fluid restrictive resuscitation can reduce FBT administration. The CLASSIC study randomized 151 adults with septic shock and showed a significant reduction of resuscitation fluid at 5 days [500 ml (IQR;0–2,500) vs. 2,000 ml (IQR; 1,000–4,100) *p* < 0.001] but no difference in total administered fluid [12,411 ml (IQR; 5,518–17,035) vs. 13,687 (IQR; 7,163–17,082) *p* = 0.45] but a trend toward lower fluid accumulation [−1,148(−2,531–235) *p* = 0.06](19). Fluid restriction also led to less AKI but no changes in rates of CRRT, respiratory support or mortality. A summary of fluid restrictive resuscitation studies in children and adults with sepsis is in Supplementary Table 1.

For pediatric studies focusing on fluid restriction it will be important to determine feasibility of implementation of a fluid restriction protocol in terms of recruitment and separation between the groups for dose of FBT. Whether restrictive FBT can present a safe, feasible alternative that positively impacts patient centered outcomes is the challenge for these studies.

## Challenges of studying interventions in pediatric sepsis: future directions

The time critical nature of recognizing and initiating management of pediatric septic shock belies the challenges of investigating FBT. Determining triggers and targets for interventions will largely rely on haemodynamic markers of shock as well as markers of impaired tissue perfusion such as hyperlactataemia. Despite the inherent difficulties, alternative interventions may prove to be safe equivalent in reversing shock and may reduce harm in terms of morbidity and mortality related to limiting excessive fluid administration. One such strategy is restrictive fluid resuscitation where early vasoactive therapy is initiated rather than repeated FBT.

### Population

Targeting children with septic shock would be necessary despite the challenges in recognizing this group early at presentation. A combination of haemodynamic indicators as well as features of organ dysfunction (altered consciousness state, tachypnoea) and tissue dysoxia (lactate elevation) are key features. A study in ANZ showed that these features are easily identifiable early and can accurately discriminate children at risk of death, albeit once admitted to an intensive care unit (30). Recent international consensus definition of sepsis severity (101) specifically recognize markers of organ dysfunction to identify high risk groups but are not designed for children. The range in age specific normal values will necessitate sophisticated trial infrastructure to ensure protocol adherence and appropriate recruitment.

### Intervention

Trials of restrictive fluid therapy will require a clinically significant separation of administered fluid volume. Vasoactive agents such as adrenaline or noradrenaline can be administered peripherally and are suitable alternate interventions. Adrenaline being more inotropic with vasoconstrictor activity would be the optimal agent. In most instances, central venous access would not be readily available and hence dilute peripheral administration would be required. The administration of peripheral adrenaline presents several issues warranting consideration. Initial titration of adrenaline would occur using non-invasive blood pressure monitoring and, in the presence of shock, would require clear pathways and ceiling doses to either enable weaning or mandate early intensive care interventions. The entry point of recruitment would need to occur when initial therapy for reversing shock are insufficient. Otherwise one risks exposing a large group of children to an intervention (or comparison therapy) that may not have been indicated thereby exposing a proportion of children to excessive therapy. The presence of septic shock and administration of 20 ml/kg of FBT and a decision to administer further resuscitation would be an example of suitable inclusion criteria.

### Outcomes

Appropriate outcomes for studies of FBT in sepsis will be an important consideration for trial designs. The desired range of outcomes should include mortality, measures of organ dysfunction, need for intensive care resources as well as outcomes specific to fluid therapy. Markers of tissue oxygenation, tissue oedema and endothelial dysfunction have also been included as secondary endpoints in current study designs. The sample size required to show a 5–10% difference in outcomes have been suggested to be up to 1,500 participants (1) which would be feasibly achieved by a multinational collaboration.

## Conclusions

FBT has been the frontline recommended therapy in sepsis management guidelines for several decades without a body of evidence supporting its appropriate use. Increasing attention has now turned to the potential consequences of excessive fluid therapy in the context of evidence suggesting harm. This has made the time ripe to further investigate the role of this long standing, fundamental intervention in pediatric sepsis. Restrictive fluid resuscitation is currently at the forefront of alternative strategies being investigated. Whether this approach is safe, feasible and effective in reducing excessive fluid therapy and can be shown to independently improve meaningful outcomes in children with septic shock remains to be seen.

## Author contributions

The author confirms being the sole contributor of this work and has approved it for publication.

### Conflict of interest statement

The author declares that the research was conducted in the absence of any commercial or financial relationships that could be construed as a potential conflict of interest.
