# Supplementary material for: Fluid Bolus Therapy in Pediatric Sepsis: Current Knowledge and Future Direction
Source: Front Pediatr. 2018 Oct 25;6:308. doi: 10.3389/fped.2018.00308 (PMC6209667; doi:10.3389/fped.2018.00308)
Supplement: Supplementary file 1 [file Data_Sheet_1.PDF]

Table 1. Studies of restrictive fluid bolus strategies in adults and children

	Population	Intervention	Comparison	Primary Outcome	Secondary Outcomes	Primary Results
**Paediatric studies**						
FiSH (24)	Children 37w-16yo with septic shock post 20ml/kg FBT	FBT 10ml/kg for persistent shock	FBT 20ml/kg for persistent shock	Study feasibility	Protocol adherence; fluid volume administration; length of hospital and ICU stay; mortality	Reduced FBT in restrictive group at 4 hours;-11.2ml/kg (95% CI –16.6 to –5.8mL/kg
SQUEEZE (23)	Children 29d-18y, fluid refractory (40ml/kg) septic shock	Early vasoactive medication	FBT up to 60ml/kg prior to vasoactive medication	Enrolment rate; protocol adherence	Assessment of protocol	Result pending
**Adult studies**						
CLASSIC (20)	153 Adults with sepsis SBP<90, lactate >4mmol/l, HR >140, post 30ml/kg FBT	Noradrenaline and 250-500ml FBT for MAP≥65mmHg	Noradrenaline and FBT targeting MAP≥65mmHg	Resuscitation volume	Fluid Balance 5d/ICU discharge; Fluid input 5d/ICU discharge; protocol violations	Reduced FBT volume by day 5 in restrictive group (-1241ml(-2043 to-439)), Reduced fluid balance
REFRESH (21)	Adults with sepsis SBP <100 post 1000ml FBT	1000ml FBT	Maintenance fluid/ early vasopressor	Total volume administered in next 6 hours	Fluid volume at 24 hours, organ support free to 28 days, mortality (90d)	Result pending
Andrews (100)	212 adults: 90% HIV + sepsis and hypotension	Sepsis protocol; FBT up to 4L of crystalloid followed by dopamine infusion	Clinician driven usual care	In hospital mortality	28 day mortality: worsening hypoxaemia; volume of FBT	Increased mortality with sepsis protocol 48% vs 33% RR 1.46 95%CI2-28
RIFTS	150 adults with sepsis and systemic inflammatory response syndrome	FBT up to 60ml/kg followed by vasopressor therapy	FBT unlimited followed by vasopressor therapy	Composite of persistent organ dysfunction and mortality (60d)	Composite of persistent organ dysfunction and mortality (7d)	Result pending

CLOVERS	Estimated 2320 patients with sepsis and hypotension	Early noradrenaline	FBT 500ml, up to 5L	90 day mortality	Organ support free days; length of stay	Result pending
